# Recruitment of low-income pregnant women into a dietary and dental care intervention: lessons from a feasibility trial

**DOI:** 10.1186/s13063-020-4142-5

**Published:** 2020-03-05

**Authors:** Amanda Rodrigues Amorim Adegboye, Paula G. Cocate, Camila Benaim, Maria Claudia da Veiga Soares Carvalho, Michael M. Schlüssel, Maria Beatriz T. de Castro, Gilberto Kac, Berit L. Heitmann

**Affiliations:** 1 Faculty of Education, Health and Human Sciences, School of Human Sciences, University of Greenwich, Park Row, London, SE10 9LS UK; 2 Nutritional Epidemiology Observatory, Department of Social and Applied Nutrition, Institute of Nutrition Josué de Castro, Rio de Janeiro Federal University, Rio de Janeiro, Brazil; 3 Department of Physical Activity Biosciences, Federal University of Rio de Janeiro, Rio de Janeiro, Brazil; 4 Department of Social and Applied Nutrition, Institute of Nutrition Josué de Castro, Rio de Janeiro Federal University, Rio de Janeiro, Brazil; 5 Centre for Statistics in Medicine, Nuffield Department of Orthopaedics, Rheumatology and Musculoskeletal Sciences, University of Oxford, Oxford, UK; 6 Research Unit for Dietary Studies at the Parker Institute, Bispebjerg and Frederiksberg Hospital, Copenhagen, The Capital Region Denmark; 7 Section for general Practice, Institute of Public Health, Copenhagen, Denmark

**Keywords:** Feasibility randomised trial, Pregnant women, Recruitment

## Abstract

**Background:**

There are difficulties in carrying out research in low-income urban communities, but the methodological challenges and suggestions on how to deal with them are often undocumented. The aims of this study are to describe the challenges of recruiting and enrolling low-income pregnant women with periodontitis to a clinical trial on vitamin D/calcium milk fortification and periodontal therapy and also to describe the patient-, study protocol- and setting-related factors related to women’s ineligibility and refusal to participate in the study.

**Methods:**

A mixed-method sequential exploratory design was applied. Qualitative and quantitative data on recruitment to a 2 × 2 factorial feasibility clinical trial were used. Eighteen women attending the health centre in a low-income area in Duque de Caxias (Rio de Janeiro, Brazil) took part in focus group discussions, and the data were thematically analysed. Quantitative data were analysed using appropriate descriptive statistics, including absolute and relative frequencies.

**Results:**

Of all referrals (767), 548 (78.5%) did not meet the initial eligibility criteria. The main reason for exclusion (58%) was advanced gestational age (> 20 weeks) at first prenatal appointment. In the periodontal examination (dental screen), the main reason for exclusion was the presence of extensive caries (64 out of 127 exclusions). Non-participation of those eligible after the periodontal examination was approximately 24% (22 out 92 eligible women) and predominantly associated with patient-related barriers (e.g. transportation barriers, family obligations, patients being unresponsive to phone calls and disconnected telephones). The study recruited 70 women with periodontitis in 53 weeks and did not reach the benchmark of 120 women in 36 weeks (58.3% of the original target). Recruitment was severely hindered by health centre closures due to general strikes. The recruitment yields were 9.1% (70/767) of all women contacted at first prenatal visit and 76.1% (70/92) of those screened eligible and enrolled in the trial. Women did not report concerns regarding random allocation and considered fortified milk as a healthful and safe food for pregnant women. Some women reported that financial constraints (e.g. transportation costs) could hinder participation in the study.

**Conclusion:**

Engagement between the research team and health centre staff (e.g. nurses) facilitated referral and recruitment, yet some pregnant women failed to participate in the study largely due to significant patient-related sociodemographic barriers and setting-related factors. Our data illustrate the complexity of overcoming recruitment and enrolment challenges for clinical trials in resource-limited settings.

**Trial registration:**

ClinicalTrials.gov, NCT03148483. Registered on 11 May 2017.

## Introduction

Difficulties in carrying out research in low-income and vulnerable urban communities are increasing due to issues of safety and setting-related factors (i.e. political instability) [1]. However, the challenges incurred with and suggestions for the solution of these difficulties are not often reported in scientific papers.

There is a wide recognition that recruitment of participants in research is a vital element for study success, particularly in clinical trials. However, an overwhelming number of clinical trials do not meet recruitment and enrolment targets [2]. A systematic review published in 2015 investigating causes of unsuccessful trial accrual found that 19% of trials had either an early termination due to recruitment failure or completion reaching only 85% of the expected enrolment target [3]. It has been estimated that approximately 86% of clinical trials fail to meet recruitment goals within their specified timeframe [3]. Moreover, the literature suggests that trial timelines can potentially be doubled beyond initially planned recruitment periods due to low participant enrolment rates [4].

Recruitment shortfalls lead to delays and increased costs with fieldwork, early trial termination and potentially failure to draw conclusions [2]. Inconclusive clinical trials have economic, scientific and political implications, as clinical and public health decisions will not be based on the best evidence possible [4–6]. There are also ethical implications when patients are exposed to potential risks and the research does not provide advances in scientific knowledge and hence related recommendations due to lack of statistical power [7]. Additionally, recruitment failure can negatively affect the motivation and engagement of stakeholders who are closely involved with the study, participants and investigators [8].

Methodological papers describe a wide range of factors which can contribute to recruitment success, including trial design and protocol development, selection of sites, staff engagement, realistic estimation of recruitment targets and study timeframe, enhanced participant contact and communication, financial incentive or compensation and convenience and reduced participation burden [9, 10]. Regardless of scientific efforts made to identify and tackle barriers and challenges with recruitment, the problem still persists. Therefore, researchers can still benefit from real-world lessons learned from the implementation of feasibility trials. These experiences can be further shared with peers working in similar settings or scenarios. Feasibility trials are paramount to test recruitment strategies, acceptability of study design and viability of eligibility criteria, thus consequently informing the progression of large-scale definitive clinical trials [11].

With the overarching aim of adding to this body of evidence, we used recruitment data from the IMPROVE feasibility trial to (1) describe the main challenges of recruiting women to the study, (2) describe most commonly observed factors related to ineligibility and non-participation of women and (3) discuss potential successful strategies for promoting recruitment and enrolment for the future full-scale clinical trial.

## Methods

The IMPROVE study was a feasibility trial on calcium and vitamin D milk fortification and periodontal therapy for improving maternal periodontal health and metabolic and inflammatory profiles. The trial was registered in the ClinicalTrials.gov database (NCT03148483) and approved by the Ethics Committee of the Maternity School of the Federal University of Rio de Janeiro-Brazil (certificate number 1.516.656). Ethical approval was granted for the quantitative and qualitative data collection and intervention delivery.

A mixed-methods sequential exploratory design was applied consisting of two phases: a qualitative followed by a quantitative phase [12]. The qualitative data were collected and analysed to assist with the study design and help explain the results found in the quantitative phase.

The study protocol has been fully described elsewhere [13]. Briefly, a 2 × 2 factorial feasibility randomised controlled trial employed four intervention groups (without cross-over). Concealed randomisation, using a mixture of permuted block sizes, stratified by smoking status was performed remotely via an online system developed by Sealed Envelope Ltd*.* Adult pregnant women with periodontitis were randomly allocated into the following groups: (1) fortified sachet with vitamin D and calcium and powdered milk plus periodontal therapy during pregnancy, (2) placebo sachet and powdered milk plus periodontal therapy during pregnancy, (3) fortified sachet with vitamin D and calcium and powdered milk plus periodontal therapy after delivery, and (4) placebo sachet and powdered milk plus periodontal therapy after delivery. Given the nature of the intervention (periodontal treatment), full blinding was not applied.

Baseline data were collected up to the second gestational trimester after checking for participant eligibility to the trial, which included a dental screening for periodontitis (T0), with follow-ups at third trimester (T1; during the course of the intervention) and 6–8 weeks postpartum (T2).

### Eligibility criteria

The trial target population included low-risk adult pregnant women, with periodontitis, attending a public prenatal care service in Rio de Janeiro, Brazil. Low-risk pregnancy was defined as not requiring management by a specialist to help ensure the best outcome for the mother and baby. The inclusion and exclusion criteria are fully described in Table 1.

**Table 1:** Eligibility criteria for the feasibility trial

Inclusion criteria	Exclusion criteria
To be included women had to:	Women were excluded if they:
Be aged ≥18 years at the time of recruitment	Had a positive diagnosis of HIV/AIDS, syphilis, psychosis, diabetes before or during pregnancy, thyroid disease or any disorder causing vitamin D hypersensitivity (e.g. sarcoidosis and other lymphomatous disorders)
Be up to 20 weeks’ gestation at 1st prenatal visit	Had lactose intolerance, milk allergy, history of kidney stones or family history of kidney stones or hyperparathyroidism
Have a positive diagnosis of periodontitis (≥ 1 tooth with at least one site with equal or more than 4 mm of clinical attachment loss [CAL] and presence of bleeding on probing); and	Were being prescribed or using antibiotics or any immune suppressants or medication affecting vitamin D/calcium metabolism or had drug or alcohol abuse
Be cognitively and physically able to complete an interview and oral examination and willing to participate, including providing blood samples	Reported consumption of equal or more than 4 servings per day of dairy products or taking vitamin D supplements more than 400 IU/day
	Had extensive dental decay (crowns of several teeth destroyed by caries) or use of fixed dental braces

### Setting

Two study sites in Duque de Caxias in Rio de Janeiro State, Brazil were initially selected for this feasibility trial. However, recruitment was conducted in one site in the Municipal Health Centre of Duque de Caxias [13]. The reasons for including only one site are presented in the “Results” section. The Municipal Health Centre provides prenatal care for low-risk pregnant women, child health programmes, as well as clinical laboratory results. The population assisted by the centre is of low income, and the majority live in the surrounding slums.

Brazil is the largest country in Latin America and the fifth largest country in terms of population and size; the population is clustered around the cities or along the coast. Rio de Janeiro and São Paulo are the most populated cities, both located in the Southeast Region of the country [14].

In the state of Rio de Janeiro, most clinical centres and hospitals are located in urban areas. Rio de Janeiro has the country’s largest contrasts in wealth, and many populations live in poverty [14, 15]. Approximately 22% of its population of six million live in slums (favelas) or in substandard housing conditions [16]. Duque de Caxias is a metropolitan city located in Rio de Janeiro State. The total population is about 900,000 inhabitants [17]. The local neonatal mortality rate is 8.9 per 1000 live births compared to 8.7 in Rio de Janeiro and 8.8 nationally [18]. The prevalence of low birth weight in Duque de Caxias is 9.2% compared to 9.2% in Rio de Janeiro and 8.5% nationally [18]. The maternal death rate in Duque de Caxias is 95.7 per 100,000 live births compared to 71.6 in Rio de Janeiro and 58.4 nationally [19]. Approximately 20% of families are covered by the Family Health Strategy, which is a federal programme to provide integrated primary health care [20] that also targets food-insecure households [21].

### Recruitment

**Fig. 1:**
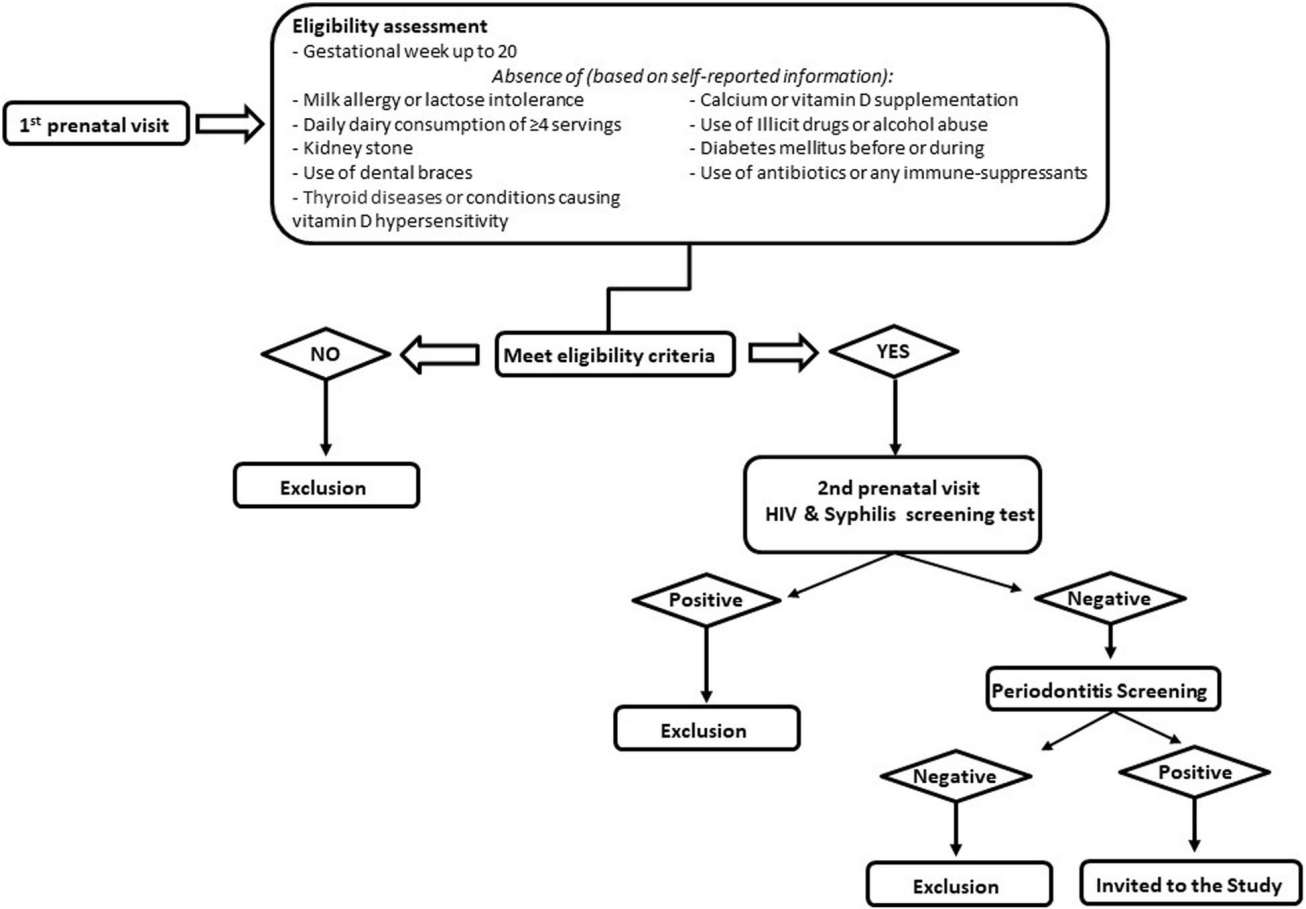
Study eligibility flow diagram. Source: Modified from Cocate et al., 2019 [13]

Staff at the prenatal clinic provided the research team with a weekly list of all pregnant women visiting the centre for the first time. In the first prenatal visit, a member of the research team approached the pregnant women, briefed them on the study objectives and procedures and invited them to participate in the study. Those initially interested in taking part in the study were asked to provide answers to a preliminary checklist for eligibility. After this, women were screened for syphilis and HIV, as part of the routine prenatal care in Brazil. Those who were preliminarily eligible and tested negative for syphilis and HIV were subsequently invited to book a dental examination for periodontitis diagnosis. Women who screened positive for periodontitis and accepted to participate were provided with an informed consent form and included in the study (Fig. 1).

The research team heavily counted on the referral by the nurses from those booking the first prenatal visit at the health centre. However, the research team also distributed flyers and hung posters about the study to promote recruitment.

### Patient and public involvement

The research team conducted a series of consultations with health care professionals, female health care users and pregnant women attending health centres in Duque de Caxias prior to the study commencement. The aim of these consultations was to understand the views of these people on the study design, assess whether the intervention would be considered relevant for the population and ask for suggestions on how the project could be implemented and also on how researchers could invite pregnant women to participate and adhere to the proposed protocol. These consultations were part of the public and patient involvement stage of the project. Individuals involved in these consultations did not provide a signed written informed consent, as these consultations were a pre-study exercise. Therefore, quotes from these informal public consultations will not be presented in this paper. The views and suggestion of the health care professionals and service users were taken into consideration when designing the study protocol.

### Data collection

In addition to the informal consultations, qualitative data on issues regarding recruitment strategy, study design and data collection were obtained prior to trial recruitment. One pilot focus group was performed in one of the eligible study sites with five non-pregnant women of similar socioeconomic conditions to those attending the health centre where the present study took place. The pilot study used a convenient sample. Then, a second semi-structured focus group was held, including 13 purposely selected pregnant women who were attending the health centre but not yet taking part in the study. Women were selected based on their pregnancy status and gestational age. The second focus group included discussion on culinary knowledge, health care practices, network and social support at Duque de Caxias territory and barriers and enablers to participation in the study. Thus, in the present study, only data on the barriers and enablers to participation and recruitment are presented.

A study recruitment-tracking log was created to document the number of women visiting the centre for the first prenatal appointment, dates of referral to the study, orientation session, periodontal examination (screening) and blood test appointments. Reasons for ineligibility, non-participation and missed appointments were also recorded.

### Data management and analysis

Quantitative data regarding recruitment were recorded in Microsoft Excel spreadsheets. For the qualitative data, focus group discussions were audio-recorded and verbatim transcribed (in Portuguese). The transcripts were analysed by three investigators (PC, CB and NHAS) to determine the themes. Each transcript was read several times, and relevant topics referring to barriers and facilitators to participation were highlighted. The data were thematically analysed. Main themes were developed from the topic groups, and appropriate theme headings and sub-headings were generated to summarise the data being presented.

**Fig. 2:**
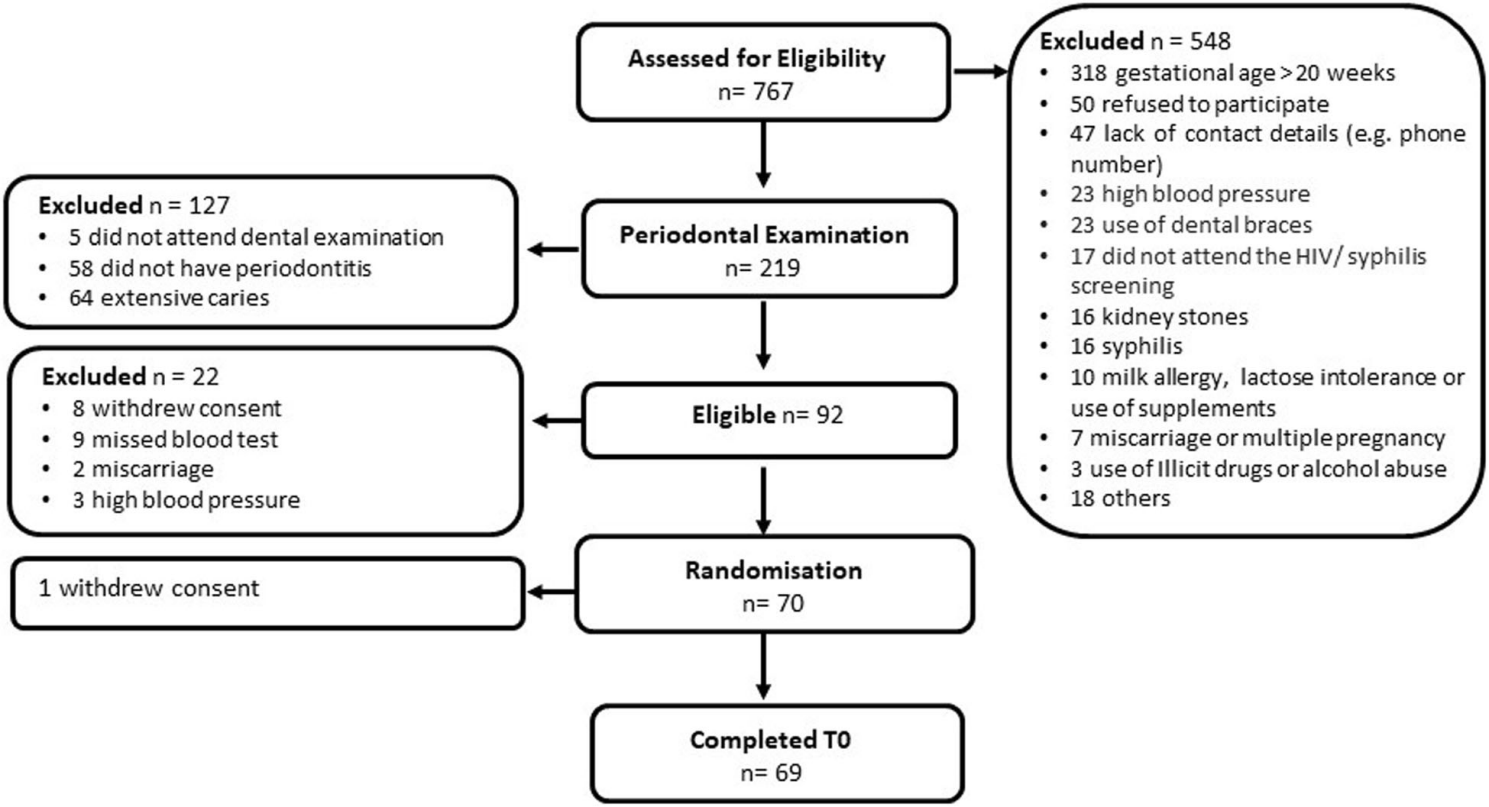
Study enrolment and exclusion flow diagram

The total number of participants recruited into the study, time for recruitment, number of invited women and number of excluded participants before and after the periodontal screening with reasons for exclusion are presented in the study flow diagram (Fig. 2). Quantitative data were analysed using appropriate descriptive statistics, including counts, percentages, mean and standard deviation (SD).

## Results

### Recruitment timeline and challenges

The participant recruitment period started at the end of April 2017 and finished at the end of May 2018. The original timeline for the recruitment was between May 2016 and January 2017. The delay in trial commencement was due to several unforeseen problems with the export of the fortified milk from Europe to Brazil, the long process for research ethical clearance and the need to re-select the study site.

The research team developed a tailored fortified milk powder for non-commercial use.^1^ Acquisition of raw material, blending, packaging and physical and chemical analysis of the products (fortified milk and plain milk) were performed in Denmark. Export of dairy products to Brazil is tightly regulated, and particularly (and unanticipated) for non-registered and non-commercial products. Given the major delays in the product export, the research team decided to change the research protocol to offer women a local commercially available milk powder and individual sachets with vitamin D3 and CAPOLAC® (source of calcium) for domestic fortification. CAPOLAC® and vitamin D3 were blended and stored in single-dose sachets. All women were provided with two daily doses of semi-skimmed milk powder (20 g) to be reconstituted in 200 ml of potable water for each serving and two sachets. Participants in the fortified intervention group received sachets with calcium and vitamin D; those in the plain milk group received placebo sachets.

The team also faced some challenges when selecting the study site. Several contacts with the local centres were made in 2015, while the research team was seeking funding and developing the study application, to obtain information on the centre prenatal logistics, monthly uptake, demographic profile, facilities and provision of dental care to pregnant women. For safety reasons (especially regarding the security of the field workers), sites involved in areas at risk of conflict and violence were excluded. The safety of centres located in slums worsened during this selection process, and it became impossible to include them as recruitment sites. Therefore, two large health centres located in Duque de Caxias, with a catchment area including deprived areas, were selected. Consent was given by the centres’ directors to conduct the trial within the centre premises with minimal interference in their routine practice. However, the senior management of the centres changed after local elections in 2016, and consent from one of the centres was withdrawn.

Between April 2017 and May 2018 (53 weeks), participant recruitment was halted several times due to strikes of health care professionals in different municipalities of Rio de Janeiro, an episode of armed robbery, local riots, and public holidays. All primary health care services in Duque de Caxias were closed for a total of 16 weeks between July and October 2017 because of general strikes. After the re-opening of the health centre in October 2017, the research team was victim of an armed robbery; both the equipment and personal belongings were stolen. Consequently, fieldwork, including the recruitment of new participants, was interrupted for 2 weeks. The health centre did not book new prenatal appointments for the week between Christmas and New Year (December 2017) or during the week of the Carnival celebration (February 2018). Therefore, recruitment was interrupted for 2 additional weeks. Furthermore, primary care centres were closed during national and local holidays (non-overlapping with strikes, Carnival and Christmas break), resulting in 4 extra days of recruitment interruption.

During the recruitment stage, Brazil was facing a period of political instability prior to and after the presidential impeachment in late 2016. The state of Rio de Janeiro also faced health care and security crises during this period. There were several riots and dragnets in Duque de Caxias, resulting in the closure of local shops and some public places for safety reasons. Although the health centre was open on these occasions, the overall number of service users attending the centre was greatly reduced.

Overall, during the 53 weeks of the enrolment period, recruitment was interrupted for approximately 21 weeks.

### Invitation and eligibility to participate in the study

In total, 767 pregnant women were approached by the research team; however, 548 women (71.5%) did not meet the initial eligibility criteria. The largest exclusion category (58%) was advanced pregnancy beyond week 20 at first prenatal visit (318 out of 548). This was followed by declined invitation (9.1%) and lack of contact details (8.6%) to invite women to attend additional assessment visits (e.g. periodontal examination), high blood pressure readings (4.2%), use of fixed braces (4.2%), non-appearance for the HIV/syphilis test (3.1%) within the study timeframe (before 20 weeks gestation), presence of kidney stones (2.9%) and presence of syphilis (2.9%). Only (1.8%) reported either milk allergy, lactose intolerance or daily consumption of vitamin D and supplements. Figure 2 provides more details on the reasons for exclusion. Reasons for declined study invitation were not recorded for all 50 declining women. Some women were limited by childcare or employment obligations.

Of those invited for the periodontal examination (dental screen, *n* = 219), 92 were eligible (42%) and 127 women were excluded. The main reasons for exclusion were the presence of extensive caries (64 out of 127, 50.4%) and absence of periodontitis (58 out of 127, 45.7%).

Non-participation of eligible women was identified after the periodontal examination. Of the women meeting the eligibility criteria (*n* = 92), 9 (9.8%) did not attend the baseline blood test, 2 (2.2%) had a miscarriage, 3 (3.2%) presented with high blood pressure and 8 (8.7%) withdrew consent. The reasons for withdrawal and missed appointments varied and included transportation barriers, family obligations, relocation to another area, changing to another prenatal care site, unresponsiveness to phone calls and disconnected telephones.

### Recruitment

In total, 70 women were randomised. One woman asked to leave the study immediately after randomisation. The recruitment yields showed that 9.1% (70/767) of all women contacted at first prenatal visit and 76.1% (70/92) of those screened eligible were enrolled into the trial.

The total study recruitment target was 120 women from two sites over 36 weeks, and the estimated recruitment rate was 1.7 participants per week per centre (120/36/2). However, only one site was involved, and 70 women were recruited (58.3% of the original target). The actual crude and net recruitment rates were 1.3 women/week/centre (70 women/53 weeks) and 2 women/week/centre (70 women/32 weeks), respectively.

The mean gestational age at recruitment was 14.3 weeks (SD 3.2). The mean maternal age and parity were 28 years (SD 5.7) and 1 birth (SD 1.2), respectively. In total, 86% of women were self-ascribed as non-white, and 87% were living with a partner.

### Barriers and facilitators

The qualitative data regarding factors which could hinder or facilitate recruitment and enrolment to the study were divided into five themes (see Table 2): study design and intervention, food myths, social support, views on prenatal care services and finance.

**Table 2:** Factors influencing recruitment and participation in the study

Themes and sub-themes	Barriers and facilitators
Theme 1: Study design and intervention	
Group allocation	• Being placed in a placebo group may decrease willingness to participate for some, but not all women • Offering delayed periodontal therapy may increase willingness to participate, as all women would receive treatment
Daily consumption of milk	• Mandatory consumption of pure milk may decrease the willingness to participate • Provision of food recipes using milk may increase willingness to participate
Milk provision	• Additional milk provision to the family including young children may increase willingness to participate
Safety	• Having milk allergy or lactose intolerance hinders participation • Milk fortification does not hinder the willingness to participate
Theme 2: Food myths	
Cultural beliefs	• Cultural beliefs regarding dairy intake during pregnancy generally do not hinder participation
Perception of healthful foods	• Consumption of milk-based foods is perceived as positive during pregnancy and lactation
Theme 3: Social support	
Emotional and informational support	• Lack of emotional and informational support may decrease the willingness to participate
Instrumental and practical support	• Lack of childcare provision decreases willingness to participate
Theme 4: Views on prenatal care	
Health care centre	• Positive attitude towards the care provided by the centre may increase willingness to participate
Health care professionals	• Good personal qualities and trust may increase willingness to participate
Theme 5: Finance	
Transportation	• Lack of money for transportation may decrease the willingness to participate
Financial compensation	• Provision of financial incentives or compensation may increase willingness to participate

In general, women did not report concerns about potential random allocation into different groups, given that all groups would benefit from the intervention. Offering a delayed intervention group instead of non-treatment, which was suggested during the informal consultations, was viewed as a positive factor towards study participation. The women also reported the interventions to be acceptable and relevant. However, women reported some resistance to drinking pure milk and suggested consuming the milk in smoothies and porridges instead. One woman stated that “If it is mixed with chocolate powder, I can have it. But it is manageable if I add it in foods, canjica^2^ and porridges”.

Women were asked whether they would share the milk with the rest of the family. Most women reported that they would share the milk with their children (*“*Yes, because they would want it and I would give the milk to my children”), but some reported that they would try to consume the milk when the children were not around (“but when it is time to drink the milk, it is not necessary to drink in front of my son. It can be consumed in the morning when they are sleeping”). One woman reported that she would “drink when the children are at school”.

No major concern regarding the safety of milk consumption was observed apart from cases when the participant had food intolerance. One of the participants commented “if there are recommendations in relation to gestation, I would take it only during pregnancy and that’s it. I would report if I have any side effect”. Positive attitudes towards consumption of fortified food were observed. One woman mentioned “the more vitamins the better”.

Regarding food myths and cultural beliefs, they reported that some foods must be avoided during pregnancy, but milk was not one of them. Women talked positively about milk consumption during pregnancy. Some believed that the consumption of milk and canjica would increase breast milk production. One of the participants said “My grandma who is old fashion used to say that you should always eat canjica, oranges with beetroot, and beans. You must have canjica”.

Lack of or limited social support was observed among women. Most women counted on emotional and informational support (e.g. advice, suggestions and information) from their mothers (“my husband works, I count on my mum”). Women who had other children often reported childcare difficulties (“I am alone, my mum only takes care of my son when I go to work. That’s all. She is my family. It is only me and God”).

Regarding their views on prenatal care, women had a positive view on the health care centre and trusted the doctors (“... I liked the treatment I received here when I had my daughter 10 years ago... I trust it”). They reported they chose the centre due to the quality of service and indications from friends or family (“My friend recommended, she said it is very good”).

Most women were unemployed, and some did not have permanent accommodation and reported living with extended family or in-laws. Most women had mobile phones, which could facilitate contact with the health centre, but limited credit to make phone calls. Cost of transport was cited as one barrier to attend the prenatal care. One woman said: “Remember to talk about the cost of transport”*.* Another woman commented that “There is no money for the ticket. (…) sometimes there is no money in the house to go to the health centre.”

## Discussion

The absolute recruitment yields were lower than expected (*n* = 70 women vs *n* = 120 women), but the recruitment rate per centre was higher than expected (an actual recruitment of 2 women/week vs the predicted rate of 1.7 women/week). A wide range of factors influencing recruitment into a dietary and dental care trial for pregnant women in a low-income area were examined. The major reason for ineligibility was gestational age above 20 weeks at the time of first prenatal care visit, which was a patient-related factor beyond our control. The initial plan was to recruit women during the first trimester to allow for early periodontal treatment initiation. The literature suggested that starting periodontal therapy after 21 weeks of gestation might be too late to reduce the inflammation that is related to the adverse pregnancy outcomes [22]. After auditing of the primary care data, the study inclusion criteria were amended to accept women at up to 20 weeks of gestation. Even though health services were free, late onset of prenatal care is common among deprived populations [23]. However, there were some external factors that might have exacerbated the delay in prenatal booking.

Rio de Janeiro faced Zika, dengue and chikungunya epidemics during the trial recruitment. Zika was declared a national public health emergency in 2015 [24]. In February 2016, the World Health Organization (WHO) declared Zika a public health emergency of international concern [25]. Although the epidemic was considered controlled in 2017, the potential causal relationship between Zika infection during pregnancy and foetal microcephaly, which was highly debated in the media, might have interfered with the early attendance of prenatal care, as some pregnant women may have restrained themselves at home. The long strikes and local instability and violence might have also contributed to the late onset of first prenatal visit in this population.

The demographic statistics of the population of pregnant women assisted by the health centre showed that the women were relatively young. Therefore, it was anticipated that applying a too-rigid definition of chronic periodontitis (which tends to develop with age) would result in a low recruitment rate. Therefore, periodontitis was defined as the presence of one or more teeth with at least one periodontal site with ≥4 mm of clinical attachment loss with the presence of bleeding on probing. The presence of bleeding on probing ensured the existence of local inflammation.

The recruitment estimation was based on a prevalence of periodontitis of 47% in low-risk pregnant women in Brazil [26]. In our study, we found 42% of eligible women with periodontitis. However, the prevalence of periodontitis in our population might have been higher, as 64 women were excluded due to extensive dental decay and were not further examined. These women might have also presented with periodontitis, which was not accounted for.

The qualitative data showed that, although women were positive to the study intervention, they faced financial and social support challenges which might have interfered with study participation. Informed by the public and patient consultations, the study protocol included a delayed periodontal treatment (after childbirth) instead of non-treatment to ensure that all participants would benefit from the study. This was sought to improve interest in taking part in the study. All women received milk and sachets (with vitamin D/calcium for the fortified group and placebo for the non-fortified group) during routine prenatal visits. In addition, all participants received milk surplus according to the number of children living in the household. We also offered financial compensation for the participants to cover transportation and meal costs.

According to the study recruitment flow diagram, there were few protocol-related factors for non-participation. Offering childcare at the health centre (e.g. a nursery) while pregnant women were attending the study visit might have prevented some withdrawal of consent during the initial study phase. However, this would have required additional staffing and venues, which were not feasible in the present study.

Several setting-related factors hindered recruitment. The initial plan was to include at least two study sites. To be eligible, health centres must assist a deprived population, be located in a relatively safe area, have available consultation rooms for the study team, offer dental care and be willing to subsidise part of the cost of the delayed periodontal treatment. Only two sites were deemed eligible, and only one agreed to participate. The health centre operates on a catchment area basis; therefore, we were not allowed to recruit women served by other health care providers to the study site.

The strikes also halted recruitment. In Brazil, it is mandatory to screen for HIV and syphilis upon prenatal care initiation and to report statistics and notify cases to health authorities. For this reason, recruitment of new patients during the strike period was not possible, and community initiatives for recruitment were not implemented. Had two sites been included, the recruitment target (*n* = 120) for the feasibility trial might have been met despite the unusual circumstances regarding political crises and strikes. However, this would have had implications for fieldwork logistics and costs. Given the additional costs incurred by the initial problems with milk import and delay of study commencement, the inclusion of an additional site located in a different municipality was not realistic.

Faced with the high levels of crime and violence in Rio de Janeiro, the safety of fieldworkers was also a concern. Fieldworkers were victims of an armed robbery and experienced near-miss events of theft. For the definitive trial, provision has to be made in the research budget for fieldworker training on how to respond to tense situations as well as insurance and a contingency fund to cover medical expenses and replacement of stolen equipment, if needed.

The present findings from this feasibility study should be interpreted in light of the following limitations. Our findings are based on experiences from researchers working at a single site located in a metropolitan region of Rio de Janeiro State. Therefore, our conclusions might not be fully generalisable to other contexts. Nevertheless, given that many countries in Latin America (such as Brazil, Colombia and Mexico) share distinguishable characteristics regarding organised crime [27], it is reasonable to assume that at least some of the issues faced by our research team might be similar to issues experienced by others at different sites, both locally and internationally. Unfortunately, evidence of these relevant obstacles to the conduct of research at primary health care centres in developing countries is lacking in the medical literature. We, therefore, encourage other researchers to follow this path, disseminating pragmatic aspects of research conduct with the aims of both improving future study design and establishing this as a good practice among the scientific community. The priority of this study was to evaluate the feasibility of recruitment, eligibility rate and acceptability of the study design and intervention and to identify barriers to enrolment to inform the large-scale trial. Contrary to most clinical trials, we wanted to design a recruitment protocol which does not maximize internal validity in detriment of external validity. Although the aim was to balance both internal and external validity, there were recruitment factors beyond the control and scope of the study. For example, we lacked resources to provide transportation to the study site; instead, we offered reimbursement for travel. Women could bring their children to the visits, but the research team could not offer support for childcare. Women reported competing priorities (e.g. care for other children), family issues (e.g. partners in prison) and problems with accommodation which resulted in missed appointments. However, the study lacked resources to assist women dealing with these issues. Also, we had to exclude women with extensive caries who were potentially eligible for the trial, as the study could not provide dental treatment for these women prior to randomisation.

Although we have conducted public consultations and collected qualitative information prior to study commencement to ensure that the study design was in line with stakeholders’ views (for example, inclusion of a delayed control group, provision of additional milk to the family and small financial compensation for the participants’ time), a participatory approach where study participants are engaged as partners and involved in all stages of the research was not applied. This could have led to a higher recruitment rate and potentially retention.

Finally, our qualitative data were based on a hypothetical invitation to a trial. What women reported they would do or intended to do could have been different from how they would react when faced with a real decision. In-depth face-to-face interviews with individuals who declined study invitation or withdrew after intervention commencement would have provided useful information for the large-scale trial. However, budget and capacity limitations prevented a detailed assessment of the characteristics of those who declined study participation.

Inequalities in metropolitan areas are growing worldwide, posing a challenge to national and local policymakers. This situation calls for actions and more research. However, the difficulties in carrying out research in vulnerable urban communities are increasing. Although many studies are being undertaken in low-income urban areas, the methodological challenges faced by researchers and potential suggestions on how to deal with such problems are not often documented.

Our data illustrate the complexity of overcoming recruitment and enrolment challenges for clinical trials in resource-limited settings. The lessons learned in this study (Table 3) on factors influencing recruitment and enrolment of low-income pregnant women will inform the delivery of a large-scale definitive trial and may benefit other researchers designing interventions on similar settings.

**Table 3:** Lessons learned from the feasibility trial regarding recruitment

• Promote more awareness of micronutrient deficiencies (calcium and vitamin D) and oral health problems during pregnancy in the centres prior to recruitment	
• Maintain a good relationship with nurses. However, more efforts should be made to engage with doctors	
• Avoid partnership with individuals and seek institutionalised collaboration in order to prevent the problem of discontinuity when individuals leave their posts	
• Prepare interdisciplinary tools and build educational support to assist the target population in dealing with barriers	
• Keep track of why people drop out, allowing timely improvements in recruitment and retention	
• Make provision in the research budget for fieldworker training on how to respond to tense situations and insurance for medical expenses and acquisition of any stolen equipment	
• Build team resilience and motivation	

## Data Availability

Results will be made publicly available; however, personal information about participants will remain confidential.

## Abbreviations

CAL: Clinical attachment loss
HIV: Human immunodeficiency virus
PT: Periodontal therapy
WHO: World Health Organization

## Footnotes

1 20 g of powdered semi-skimmed milk of a commercial brand available in Brazil to be mixed with a 2 g sachet, containing calcium (CAPOLAC 500 mg) and vitamin D3 (500 IU).

2 Brazilian traditional sweet dish made with white corn, milk, nuts, coconut and cinnamon. It is a dairy-based dish.
